# Comparison of Intravertebral Clefts between Kümmell Disease and Acute Osteoporotic Vertebral Compression Fracture: A Radiological Study

**DOI:** 10.1111/os.13025

**Published:** 2021-09-15

**Authors:** Zhenzhong Chen, Chao Lou, Weiyang Yu, Dengwei He

**Affiliations:** ^1^ Department of Orthopedics 5^th^ Affiliated Hospital, Lishui Municipal Central Hospital, Wenzhou Medical College Lishui China

**Keywords:** Acute osteoporotic vertebral compression fracture, Intravertebral cleft, Kümmell disease, Radiological features

## Abstract

**Objective:**

The aim of this study was to compare the radiological features of intravertebral clefts (IVC) between Kümmell disease (KD) and acute osteoporotic vertebral compression fracture (OVCF).

**Materials and Methods:**

This is a retrospective study. A total of 79 patients with IVC from January 2014 to December 2018 were included in this study. There were 22 men and 57 women, with an average of 73.5 years. Based on the exact time interval from injury to treatment and the pathological examination results, the patients were divided into KD group (44 patients) and acute OVCF group (35 patients). The two groups were compared by the margin sclerosis of IVC, vertebra and pedicle ossification, stress fracture of the spinous process, paravertebral callus, the shape of IVC, cleft in the adjacent disc, and flatness of IVC's margin from plain radiographs and computed tomography (CT). The two groups were compared by the IVC content, double‐line sign, and signal of fracture vertebral from their magnetic resonance imaging (MRI).

**Results:**

There were no significant differences in sex, age, and fracture distribution between the KD group and the acute OVCF group. IVC was present in both the KD group and the acute OVCF group. Six radiological features were only present in the KD group, including sclerosis of the cleft margin (95.5%, 42/44), ossification of the fractured vertebrae (100%, 44/44), ossification of the pedicle (31.8%, 14/44), double‐line sign (27.3%, 12/44), stress fracture of the spinous process (13.6%, 6/44), and even formation of paravertebral callus (18.2%, 8/44). Although there were statistical differences in the other four radiological features of content of IVC (*P* = 0.02), cleft sign in adjacent intervertebral disc (*P* < 0.01), margin of IVC (*P* = 0.02), and the shape of IVC (*P* = 0.01) between the KD group and acute OVCF group, these characteristics could be found in both groups.

**Conclusion:**

IVC could present in patients with both KD and acute OVCF; however, we found that marginal cleft sclerosis, vertebral and pedicle ossification, double‐line sign, spinous process fracture, and formation of paravertebral callus are unique radiological features of KD and could be used for differentiation of KD from acute OVCF with IVC.

## Graphical abstract

Intravertebral clefts (IVC) could present in patients with both Kümmell disease (KD) and acute osteoporotic vertebral compression fractures (OVCF), which leads to challenges in the differentiation of clinical diagnosis. We found that there were some imaging features that were unique to KD (Fig. 1) but not in acute OVCF (Fig. 2).

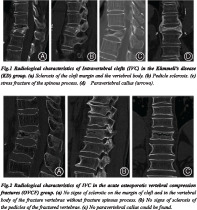

## Introduction

Kümmell disease (KD) was first described by Dr. Hermann Kümmell in 1891 when it was defined as a clinical condition in which patients develop a painful progressive angular kyphosis as a result of a delayed vertebral body collapse after minor spinal trauma. The main cornerstones of KD are a history of trauma with a negative X‐ray investigation, an asymptomatic period, and a recurrence of symptoms that result in painful kyphosis deformity of the affected spine^1^. Multiple terms have been used for describing a similar phenomenon, including delayed post‐traumatic vertebral osteonecrosis, intravertebral pseudarthrosis, intravertebral vacuum cleft, delayed vertebral collapse, and nonunion of compression fracture^2^. However, none of these terms are precise enough for a specific representation of the disease; thus, in order to maintain consistency in this article, we will refer to this pathology as Kümmell disease.

Intravertebral cleft (IVC) of senile osteoporosis used to be considered as the unique characteristic of KD^3^, ^4^. Still, increasing evidence has shown that IVC is also present in some of the acute osteoporotic vertebral compression fractures (OVCF)^5^. Most patients with OVCF can be successfully managed with conservative treatments; percutaneous vertebroplasty (PVP) and kyphoplasty (PKP) have become widely accepted as a treatment for OVCF that could not be treated with conservative treatments^6^. Yet, for the KD patients, conservative treatment is generally not recommended and the treatment is not limited to PVP and PKP as these surgeries have a higher failure rate than in acute OVCF^7^, ^8^, ^9^. The treatment options for KD continue to raise controversies; still, it is first necessary to assess the patient's basic condition, the stability of fracture, and neurological deficits, after which PVP and PKP, posterior internal fixation, anterior reconstruction or posterior osteotomy should be performed^10^, ^11^, ^12^. Since the treatments for KD and acute OVCF are different, it is necessary to differentiate diagnosis before decision‐making.

KD is also known as avascular necrosis after osteoporotic vertebral compression fracture^13^. The exact time that injury occurs is a critical clue for differentiation of the diagnosis for KD and acute OVCF. Pathologically, KD is a chronic process from fracture to delayed fracture healing. Although in most of the cases, when fracture happens or fracture healing is delayed, the pain that the patients experience can point to some evidence. Nevertheless, it is sometimes difficult for the elderly population to recall the details related to the injury they experienced. In this scenario, it is difficult to differentiate KD from acute OVCF based on patients' chief complaint only, which highlights the importance of radiological evidence that would improve diagnosis.

We hypothesized that due to the different history time and pathogenesis of the two diseases, the two should show different imaging characteristics. Herein, we propose a reliable method for differentiation of diagnosis between KD and acute OVCF based on the radiological features of IVC. The aim of this study is to: (i) confirm the presence of IVC in some acute OVCF; (ii) summarize the imaging characteristics of IVC in KD and acute OVCF; and (iii) compare the imaging features of IVC between IVC and acute OVCF to find the key points of the differential diagnosis of the two.

## Materials and Methods

### Inclusion and Exclusion Criteria

Inclusion criteria: (i) aged 60 years or older; (ii) fragility fracture of thoracic or lumbar vertebra (without trauma or with minor trauma, as tumble or sprain); (iii) underwent surgical intervention; (iv) IVC in the vertebral body could be found by CT imaging; and (v) complete radiological information including X‐ray, CT, and MRI could be achieved.

Exclusion criteria: (i) burst fracture of a thoracic and lumbar vertebra; (ii) pathological fracture due to infection or malignancy; (iii) with adjacent vertebral fractures; (iv) incomplete radiological information; (v) younger than 60 years old; (vi) cannot recall the details about the injury; (vii) with no history of injury; and (viii) underwent conservative treatment. The study was approved by the institutional review board and the ethics committee of our hospital.

### Patient Enrollment

This was a retrospective, comparative study of patients with IVC in Lishui Municipal Central Hospital from January 2014 to December 2018. Every included patient also needed to recall the details on the exact time interval from injury to treatment. According to the interval time, patients with an interval time of more than 3 weeks were assigned to the KD group, and those with less than 3 weeks were assigned to the acute OVCF group. A biopsy for pathological examination was performed in all patients during surgery. Patients with signs of necrotic bone tissue with marked fibrous tissue hyperplasia and newborn bone formation were categorized as KD patients, whereas acute OVCF categorization was based on a pathological finding of remote hemorrhage, obvious local granulation tissue hyperplasia, some fibroblast proliferation, and occasionally a small amount of fibrous callus. Patients with inconclusive pathological reports or inconsistent pathological reports were excluded.

**Fig 1:**
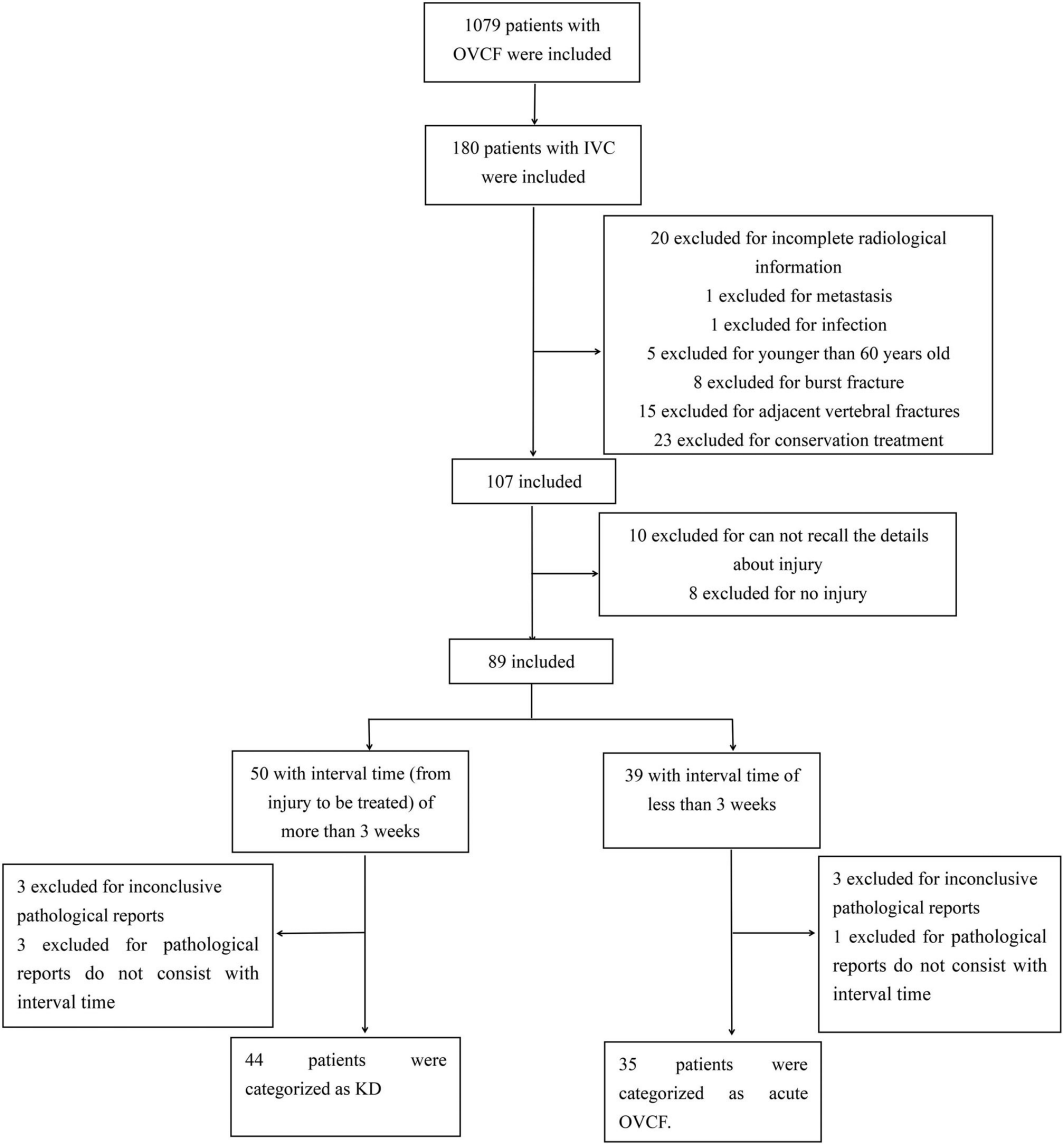
Flowchart of patients in the study.

According to the interval time from injury to treatment, 50 patients were categorized as KD and 39 patients as acute OVCF. Combined with pathological results, six patients in the KD group and four patients in acute OVCF group were excluded for inconclusive pathological reports. Finally, 44 patients were diagnosed as KD, whereas 35 patients were diagnosed as acute OVCF (Fig. 1).

### Radiology Technique

The plain radiographs were scanned using Raycus Direct Digital Radiography System (Carestream Health Inc., Rochester, NY). Imaging parameters for plain radiographs were as follows: lumbar anteroposterior are 75–85 kV, 30–55 mAs; lateral images are 85–90 kV, 15–25 mAs.

The CT images were scanned using dual source CT (SOMATOM Force, Siemens Healthineers，Forchheim，Germany). Imaging parameters for CT scanning were as follows: the tube voltage was 120 kV, the CARE DOSE 4D was applied, and the reference mAs was set to 300. Reconstruction parameters: bone window and soft tissue window were reconstructed, layer thickness and layer spacing were 3 mm; thin layer thickness,1.0 mm; layer spacing, 0.75 mm; matrix, 512 × 512; pitch 0.8; collimator width, 192 × 0.6 mm; matrix, 512 × 512; field, 30 × 30–35 × 35cm.

The MRI images were scanned using Magnetic Resonance Imaging System (MAGNETOM Aera 1.5T, Siemens Healthineers，Forchheim，Germany). Imaging parameters for MRI scanning were as follows: the scanning parameters were conventional sagittal (SAG) T1WI, T2WI, and FST2WI lipid suppression, and conventional transverse (TRA) T2WI.

### Radiographic Evaluation

#### Interobserver Reliability

The results of the radiological examination of all patients were independently reviewed by two experienced radiologists. In case of disagreement between the two investigators, a third investigator was involved in the decision‐making process. A final decision was reached by mutual consensus. Kappa reliability coefficients were used to assess interobserver reliability.

#### Outcome Measures of Plain Radiographs and Computed Tomography

**Fig 2:**
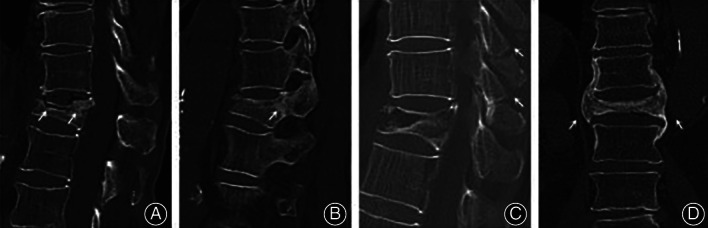
Radiological characteristics of IVC in the KD group. (A) Sclerosis of the cleft margin and the vertebral body. (B) Pedicle ossification. (C) Stress fracture of the spinous process. (D) Paravertebral callus (arrows).

**Fig 3:**
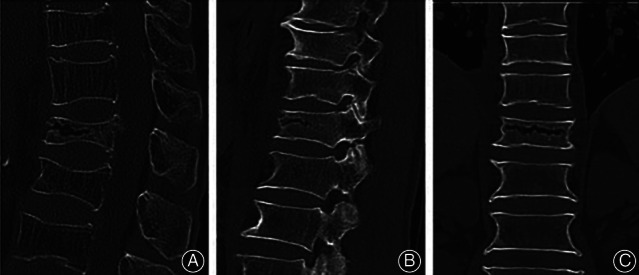
Radiological characteristics of IVC in the acute OVCF group. (A) No signs of sclerosis on the margin of cleft and in the vertebral body of the fracture vertebrae without fracture spinous process. (B) No signs of ossification of the pedicles of the fractured vertebrae. (C) No paravertebral callus could be found.

**Fig 4:**
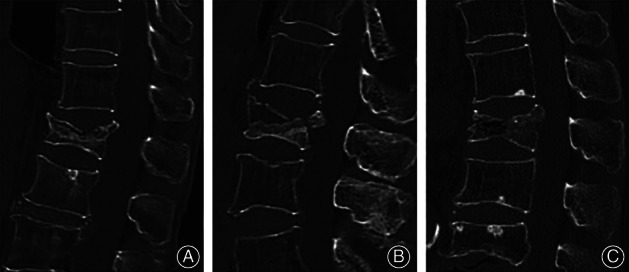
Demonstration of different shapes of IVC. (A) Linear type: IVC is evenly and continuously distributed in the vertebral body. (B) Triangular type: displayed in the anterior column of the vertebrae with triangular distribution. (C) Irregular type: unevenly and irregular distribution.

Margin sclerosis of IVC was defined as a layer of bone sclerotic change around the IVC. Vertebra and pedicle ossification was defined as an area of ossification (CT value more than 200 HU) that could be discovered in vertebra or pedicle. Paravertebral callus was defined as reactive callus formation surrounding the site of the fracture; the osteophyte was excluded. The IVC's margin was categorized as flat or uneven. Stress fracture of the spinous process was defined as a spinous process fracture of an injured vertebra and adjacent vertebra. A cleft in the adjacent disc was defined as any perceptible shape of a cleft in an adjacent disc. Shapes of IVC were categorized into three patterns: linear type (IVC was evenly and continuously distributed in the vertebral body), triangular type (IVC in the anterior half of the vertebral body with a triangular distribution), and irregular type (IVC was uneven or it had several lines but no continuous distribution).(Figs 2, 3, 4).

#### Outcome Measures of Magnetic Resonance Imaging (MRI)

**Fig 5:**
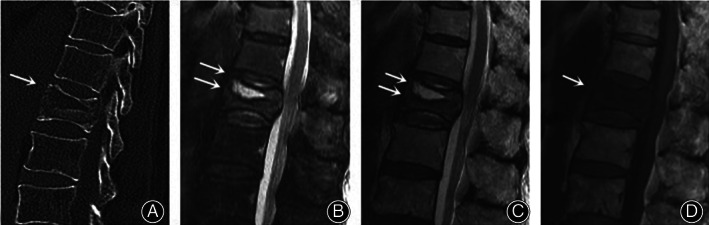
(A) CT image of a 78‐year‐old man showing an IVC in L1. (B) Sagittal STIR image and (C) Sagittal T2‐weighted MRI showing a high linear signal with surrounding low intensity is visible (“double line sign”; arrows). (D) Sagittal T1‐weighted MRI showing a low signal in L1.

The content of IVC was categorized into gas and liquid; if the content was mixed with gas and liquid, it was categorized as a liquid. Gas was defined as low signal intensity on T1‐weighted and short‐time inversion recovery (STIR) magnetic resonance images. The liquid was defined as low signal intensity on T1‐weighted magnetic resonance images, with high signal intensity on STIR images. A double‐line sign was identified as a peripheral zone of low intensity surrounding the band of the high intensity on STIR images (Fig. 5). Signal of vertebral fracture was divided into low signal intensity and high signal intensity of T1‐weighted and STIR images.

### Statistical Methods

All demographic and clinical data were collected and expressed as mean ± SD for descriptive data. Differences between two groups were assessed with the use of Student t‐test or nonparametric tests. Radiological features between the two groups were compared using χ^2^ test. Significance was set at a *P*‐value of 0.05. Interobserver reliability in the radiological features was evaluated using kappa coefficients (strength of agreement defined as <0 poor, 0.01–0.2 slight, 0.21–0.4 fair, 0.41–0.6 moderate, 0.61–0.8 substantial, and 0.81–1 almost perfect). The SPSS statistical program (SPSS, Chicago, IL) version 17.0 was used for statistical analysis.

## Results

### Patients' Demographic Data

Forty‐four patients with an average age of 75.3 ± 8.2 were diagnosed as KD, whereas 35 patients with an average age of 72.3 ± 8.8 were diagnosed as acute OVCF (Fig. 1). The bone mineral density (BMD) of the KD group (−3.11 ± 0.67) was significantly lower than that of the acute OVCF group (−2.59 ± 0.71, *P* = 0.001). There were no differences in the gender, body mass index (BMI), and fracture distribution between the two groups (*P* > 0.05; Table 1).

**TABLE 1:** Summary of patient background data

		KD group (n = 44)	Acute OVCF group (n = 35)	*P*
Age (yrs)		75.3 ± 8.2	72.3 ± 8.8	0.13
Gender (M/F)		12/32	10/25	0.90
BMI (kg/m^2^)		25.9 ± 2.9	25.2 ± 3.5	0.34
BMD (T‐score)		−3.11 ± 0.67	−2.59 ± 0.71	0.001
Fracture distribution	T_7_	1 (2.3%)	0	0.55
	T_8_	0	1 (2.9%)	
	T_9_	1 (2.3%)	2 (5.7%)	
	T_10_	4 (9.1%)	1 (2.9%)	
	T_11_	9 (20.5%)	6 (17.14%)	
	T_12_	10 (22.7%)	14 (40.0%)	
	L_1_	14 (31.8%)	7 (20.0%)	
	L_2_	3 (6.8%)	3 (8.6%)	
	L_3_	2 (4.5%)	1 (2.9%)	

BMD, Bone mineral density; BMI, Body mass index; IVC, Intravertebral cleft; KD, Kümmell disease; OVCF, Osteoporotic vertebral compression fractures.

### Unique Radiological Features Only Presented in the KD Group

Among the 10 interpreted radiological features, we identified six as only present in the KD group and shown as follows.

#### Sclerosis of the Cleft Margin

Sclerosis of the cleft margin was found in 95.5% (42/44) of the IVC in the KD group; however, this radiological feature was not presented in the acute OVCF group (0/35, *P* < 0.01) (Table 2).

**TABLE 2:** Unique radiological features only presented in the KD group but not in acute OVCF

Imaging findings	KD (n = 44)	Acute OVCF (n = 35)	*P*
Cleft margin sclerosis	42 (95.5%)	0 (0.0%)	<0.01
Vertebral ossification	44 (100%)	0 (0.0%)	<0.01
Pedicle ossification	14 (31.8%)	0 (0.0%)	<0.01
Double‐line sign	12 (27.3%)	0 (0.0%)	<0.01
Stress fracture of spinous process	6 (13.6%)	0 (0.0%)	<0.01
Paravertebral callus	8 (18.2%)	0 (0.0%)	<0.01

IVC, Intravertebral cleft; KD, Kümmell disease; OVCF, osteoporotic vertebral compression fractures.

#### Ossification in the Vertebra and Ossification in the Pedicle

We also found the radiological feature of ossification in the vertebrae was only present in the KD group, which was present in 100% (44/44) of patients. For the pedicles, we found 31.8% (14/44) of the KD patients presented features of ossification. The two radiological feature was not presented in the acute OVCF group (*P* < 0.01) (Table 2).

#### Stress Fracture of the Spinous Process

Stress fracture of the spinous process is a result of instability, and occurred in 13.6% (6/44) of patients from the KD group and none in the acute OVCF group (0/35, *P* < 0.01) (Table 2).

#### Paravertebral Callus Formation

The incidence of paravertebral callus formation was not high, but only occurred in the KD group (18.2%, 8/44) and none in the acute cases (0/35, *P* < 0.01) (Table 2).

#### Double‐Line Sign

According to the MRI, the double‐line sign was present in about 27.3% (12/44) of patients in the KD group. However, they were not present in any of the OVCF acute cases (0/35, *P* < 0.01) (Table 2).

### Common Radiological Characteristics of IVC in Both KD and Acute OVCF Groups

In addition to the above six radiological characteristics, the other four radiological characteristics were found in both KD and OVCF groups, but there was a statistical difference between the two.

#### Content of IVC

In both groups, MRI signals of injured vertebral bodies showed low signal intensity on T1‐weighted images and high signal intensity on STIR images. Gas could be inspected in the IVC for both groups, with about 54.5% (24/44) for the KD group and 28.6% (10/35) for the acute OVCF group (*P* = 0.02) (Table 3).

**TABLE 3:** Common radiological characteristics of IVC in both KD and acute OVCF groups

Imaging findings	KD (n = 44)	Acute OVCF (n = 35)	*P*
Content of IVC			0.02
Liquid	20	25	
Gas	24	10	
Cleft sign in adjacent intervertebral disc			<0.01
Yes	26	9	
No	18	26	
Margin of IVC			0.02
Flatness	33	17	
Uneven	11	18	
Shape of IVC			0.01
Linear type	27	10	
Triangular type	7	9	
Irregular type	10	16	

IVC, Intravertebral cleft; KD, Kümmell disease; OVCF, osteoporotic vertebral compression fractures.

#### Cleft Sign in Adjacent Intervertebral Disc

The IVC in the vertebral body had significantly more cleft in the intervertebral disc in the KD group compared to the acute OVCF group (59.1% *vs* 25.7%, *P*<0.01) (Table 3).

#### Shape of IVC and margin of IVC

Regarding the shape of IVC, the margin of IVC was flatter in the KD group (75% *vs* 48.6%, *P* = 0.02). Meanwhile, liner type (61.4%, 27/44) of IVC was more commonly observed in the KD group, while irregular type (45.7%, 16/35) was most commonly seen in the IVC of the acute OVCF group (*P* = 0.01) (Table 3).

### Interobserver Reliability

The kappa value of interobserver reliability was 0.949 for the presence of the sclerosis of the cleft margin, 0.923 for vertebral and pedicle ossification, 0.78 for a stress fracture of the spinous process, 0.771 for paravertebral callus, 0.782 for the shape of IVC, 0.673 for the flatness of IVC's margin, 0.746 for a cleft in an adjacent disc, 0.847 for the content of IVC.

## Discussion

KD is an OVCF‐related complication. The disease can occur several months after the initial spinal injury, and it is characterized by delayed development, which makes it different from common OVCFs. Multiple terms have been used for describing KD; however, to maintain consistency throughout this article, we referred to this pathology as KD^14^.

Compared with acute OVCF with IVC, KD is more complicated for treatment and has higher failure rates during percutaneous vertebroplasty or kyphoplasty. According to Lee *et al*.^15^, the treatment of KD is more prone to failure due to injections of Polymethyl‐methacrylate (PMMA) into a cystic cavity that are believed to have far less interdigitation with the surrounding bone compared to an injection into a partially intact trabecular bone. PMMA cement in vertebroplasty thus merely functions as space‐occupying material without any mechanical interlock or biocompatibility. Therefore, there is the potential for dislodgment or fragmentation leading to a further kyphotic deformity. Heo *et al*.^16^ investigated the incidence rate, characteristics, and predisposing factors associated with re‐collapse of the same vertebrae after PVP and concluded that the most important predisposing factor for re‐collapse was preoperative osteonecrosis.

The differences in the treatment of KD and acute OVCF highlight the importance of diagnosis differentiation between the two^6^, ^7^, ^10^, ^11^, ^12^. Due to the different pathogenesis of KD and acute OVCF, the most vital clue to the differential diagnosis is the detailed history of the injury. Yet, from a clinical point of view, doctors are not only facing a simple task of distinguishing between fresh OVCF and old OVCF, since osteoporotic vertebra fractures in the elderly are often caused by minor trauma, such as bending, twisting, or even coughing, and quite often these patients cannot recall the injury very well. Furthermore, it is not easy to distinguish whether it's KD or acute OVCF merely through imaging, because the MRI signals of fracture vertebral body all show low signal intensity on T1‐weighted images and high signal intensity on STIR images. Therefore, it is essential to diagnose KD and acute OVCF based on other radiological features.

In the present study, we identified six radiological features that were only found in IVC of the KD group. Marginal sclerosis of IVC (95.5%) and ossification around IVC of the vertebral body (100%) are the two most essential features for differential diagnosis. Although CT examination of acute OVCF can also sometimes reveal an increase in CT value around the fracture, careful analysis of the images shows that it is caused by trabecular bone accumulation around the fracture site. However, these two characteristic signs have still not received enough attention in clinical practice so far. The other four features, including ossification in the pedicles (31.8%), double‐line sign (27.3%), stress fracture of the spinous process (13.6%), and paravertebral callus formation (18.2%), which were only present in the IVC of KD, could only be used to assist diagnosis due to their low incidence. KD is usually defined as delayed fracture union with necrosis of the vertebral body, and bone hyperplasia and paravertebral callus formation were found around IVC in the KD group. As a result, these five distinctive radiological features can help to distinguish from the IVC of acute OVCF.

Meanwhile, some features were present in both groups, including the content of IVC, the flatness of the edge of IVC, and the cleft in the adjacent intervertebral discs^17^. These features were not unique for KD patients; however, the incidence of these characteristics was significantly different. For example, the majority of IVC contained gas in KD, and liquid in the group of acute OVCF. We found that the shape of IVC in the KD group was linear or triangular, while IVC in the acute OVCF was irregular. These features could also be helpful for differentiation between the two.

Our results could be useful for differential diagnosis and decision‐making before surgery. For instance, those with severe marginal sclerosis of IVC, PVP, or PKP should be reconsidered since PMMA has far less interdigitation with the surrounding bone, which in turn has a higher possibility of bone cement displacement. Additionally, for those IVC combined with stress fractures of the spinous process, which suggests instability of the segment, the application of internal fixation should be considered.

Our study has some limitations that need to be pointed out. First, the study was retrospectively designed, which is the main limitation. Second, the inclusion criteria and exclusion criteria were strict, and some patients with incomplete radiological information may have been excluded. Future studies are required to further the understanding of the underlying mechanism of the IVC formation in acute OVCF.

### Conclusion

Increasing evidence has shown that IVC could be present not only in patients with KD but also in some patients with acute OVCF, thus highlighting the importance of diagnosis differentiation between the two. Our study found that IVC in patients with KD had exclusive radiological features including cleft margin sclerosis, vertebral and pedicle ossification, double‐line sign, spinous process fracture, and paravertebral callus. These radiological features have never before been reported and could be useful for the differentiating diagnosis between KD and acute OVCF when IVC is found in the vertebral body.
